# Understanding the development of Th2 cell-driven allergic airway disease in early life

**DOI:** 10.3389/falgy.2022.1080153

**Published:** 2023-01-10

**Authors:** Beatriz León

**Affiliations:** Department of Microbiology, University of Alabama at Birmingham, Birmingham, AL, United States

**Keywords:** Th2 cells, infants, LPS (lipopolysaccharide), dendritic cells, IL-12 cytokine, T-bet, IL-2 cytokine, GM-CSF

## Abstract

Allergic diseases, including atopic dermatitis, allergic rhinitis, asthma, and food allergy, are caused by abnormal responses to relatively harmless foreign proteins called allergens found in pollen, fungal spores, house dust mites (HDM), animal dander, or certain foods. In particular, the activation of allergen-specific helper T cells towards a type 2 (Th2) phenotype during the first encounters with the allergen, also known as the sensitization phase, is the leading cause of the subsequent development of allergic disease. Infants and children are especially prone to developing Th2 cell responses after initial contact with allergens. But in addition, the rates of allergic sensitization and the development of allergic diseases among children are increasing in the industrialized world and have been associated with living in urban settings. Particularly for respiratory allergies, greater susceptibility to developing allergic Th2 cell responses has been shown in children living in urban environments containing low levels of microbial contaminants, principally bacterial endotoxins [lipopolysaccharide (LPS)], in the causative aeroallergens. This review highlights the current understanding of the factors that balance Th2 cell immunity to environmental allergens, with a particular focus on the determinants that program conventional dendritic cells (cDCs) toward or away from a Th2 stimulatory function. In this context, it discusses transcription factor-guided functional specialization of type-2 cDCs (cDC2s) and how the integration of signals derived from the environment drives this process. In addition, it analyzes observational and mechanistic studies supporting an essential role for innate sensing of microbial-derived products contained in aeroallergens in modulating allergic Th2 cell immune responses. Finally, this review examines whether hyporesponsiveness to microbial stimulation, particularly to LPS, is a risk factor for the induction of Th2 cell responses and allergic sensitization during infancy and early childhood and the potential factors that may affect early-age response to LPS and other environmental microbial components.

## Introduction

Allergic diseases are chronic inflammatory disorders caused by aberrant immune reactions to harmless foreign proteins called allergens. Allergic diseases can affect the respiratory tract, skin, and digestive system, causing airway allergy, atopic dermatitis, and food allergy. This review focuses on respiratory allergies, which are induced by exposure to airborne allergens. The most common aeroallergens are pollen, fungal spores, house dust mites (HDMs), and animal allergens. Respiratory allergies include allergic rhinitis and asthma, which affect the upper and lower airways, respectively. Inflammation triggered by exposure to allergens in the upper respiratory tract causes sneezing, congestion, and itchy nose, mouth, and eyes. Inflammation in the lower respiratory tract in patients with asthma can cause breathing problems and obstruct airflow when the airways swell and narrow and produce excess mucus.

Allergic respiratory disorders are increasingly prevalent in the developed world and are the most common chronic immunological diseases affecting children (1, 2). Approximately 7% of the population of the United States suffers from respiratory allergies. Among children, the frequency is higher, with around 10% of children affected by allergic disorders of the respiratory tract, of which approximately 6% have asthma (3–6). In particular, allergic airway disorders often have an early onset and usually develop before the age of 5 years and then persist throughout life (7–9). Current trends predict that pediatric respiratory allergy incidence rates will continue to rise, particularly in developed countries (4, 5, 10), providing further evidence that lifestyle change associated with urbanization and industrialization is contributing to this increase in allergic airway disease in children.

The characteristic pattern of inflammation in the airways of children with allergy is mediated by type 2 immune responses mainly regulated by T helper 2 (Th2) and type 2 T follicular helper (Tfh2) cells. Tfh2 cells secrete the cytokines interleukin (IL)-4, IL-21, and IL-13 in B cell follicles, which promotes the production of immunoglobulin E (IgE) and IgG1 by B cells (11, 12), leading to the activation of mast cells and basophils (13). Th2 effector cells secrete IL-4, IL-13, IL-5, and IL-9 upon allergen stimulation (14, 15), promoting inflammation and tissue remodeling by inducing the infiltration and activation of eosinophils (16) and excessive secretion of mucus (17). A fundamental characteristic of the development of airway inflammation upon allergen contact is that it requires an initial exposure to allergen or “*sensitization*” that does not necessarily cause symptoms or pathology. However, once a person becomes “sensitized” to an allergen, they will develop pathology following secondary or subsequent allergen exposures or “c*hallenges*.” During this initial sensitization phase, allergens trigger an immune reaction that ultimately activates conventional dendritic cells (cDCs) to prime allergen-specific CD4^+^ T cells with a Th2-like cytokine profile. The presence and persistence of these allergen-specific T cells after initial priming marks the predisposition to develop allergic responses, as these T cells can be subsequently reactivated upon re-exposure to the same inhaled allergen, causing their migration to the airways, where they locally produce Th2 cytokines (15, 18). Accumulation of Th2 effector cells in the lungs ultimately stimulates the hallmarks features of allergic inflammation, such as tissue swelling, mucus hypersecretion, and bronchial hyperresponsiveness (14).

An accumulating piece of evidence has shown that during early childhood, there is a predisposition to prime Th2-biased immune responses compared to later in life (19–21). These data suggest that environmental factors specifically impacting during a time window in early life would instigate the initial priming of aeroallergen-specific T cells toward a Th2-like cytokine profile, thus promoting allergic sensitization. Furthermore, lifestyle changes in developed countries are contributing to this trend (5, 10). Living in developed countries is associated with industrialization, urbanization, and changes in hygiene habits. The most hygienic environment and practices apply particularly to infants and young children. These changes lead to a restriction in exposure to inhalant dust-related microbial products during the first years of life. In particular, a number of epidemiologic studies have drawn attention to decreased exposure to inhalant house-dust bacterial endotoxins (lipopolysaccharides, LPS) and endotoxin-contaminated aeroallergens in childhood and the risk of developing Th2-driven, allergen-induced airway inflammation (22–30). This review discusses the mechanisms that promote and prevent Th2 cell immunity—especially highlighting the diverse signals elicited by the environmental allergens that lead to the activation of different cells in the exposed tissue, including epithelial/stromal cells, monocyte subsets, and innate lymphoid cell group 2 (ILC2). It further analyzes how signals derived from these cells are ultimately integrated by cDCs, particularly type 2 cDCs (cDC2s), to program their function to induce or prevent Th2 cell responses. Moreover, this review examines the fundamental role of endotoxin/LPS contained in airborne allergens in preventing allergen-specific T cell priming toward a Th2 cell phenotype and, thus, in preventing new allergen sensitizations. Finally, it analyzes the mechanisms that promote a state of hyporesponsiveness to endotoxins/LPS during early childhood, favoring Th2 cell sensitization to aeroallergens with low endotoxin contamination and ultimately contributing to increased susceptibility to developing respiratory allergic diseases in early life.

## Mechanisms that promote Th2 cell priming by dendritic cells

cDCs are the principal antigen-presenting cells (APCs) to initiate CD4^+^ T cell activation and proliferation by presenting protein-derived peptides on major histocompatibility complex (MHC) molecules (also known as “signal 1”) and by providing potent co-stimulatory signals through the engagement of CD28 expressed by the T cells (also known as “signal 2”). In addition, cDCs provide polarizing cytokines that determine the differentiation of naïve CD4^+^ T cells into the various effector T helper (Th) subsets (also known as “signal 3”). For example, cDC-derived IL-12 induces Th1 cell differentiation by promoting interferon-γ (IFNγ) production and expression of the Th1 cell-associated transcription factor T-bet (also known as TBX21) (31–34). Transforming grow factor-β (TGFβ) induces the transcription factor forkhead box P3 (FOXP3) and thus drives T regulatory T (Treg) cell differentiation (35). But, the presence of TGFβ along with IL-6 during T cell priming promotes the expression of the retinoic acid receptor-related orphan receptor-γt (RORγt; also known as RORC) that drives Th17 cell differentiation, thus subverting differentiation of Treg cells (36, 37). IL-23 then maintains the Th17 phenotype by stabilizing RORγt, thereby allowing Th17 cells to release their effector cytokines (38). Since IL-12, TGFβ, IL-6, and IL-23 are cytokines produced by cDCs in response to pathogen or danger recognition (39–42), it is believed that cDCs provide all three signals for the differentiation of Th1, Th17, and induced Treg responses.

Although many studies have made clear that cDCs are required for the induction of Th2 cell responses (15, 18, 43–53), an equivalent cDC-derived cytokine or “signal 3” that induces Th2 cell differentiation has not been found. Instead, early studies proposed that Th2 cell differentiation may be controlled by the strength of the “signal 1” delivered through the T cell receptor (TCR). This includes the affinity of the interactions between MHC and TCR molecules and the amount of antigen. This hypothesis was initially based on *in vitro* studies showing that weak TCR signaling as a result of stimulation with low doses of antigen or low-affinity peptides favored IL-4 over IFNγ production (54–58). And although *in vivo* studies using adoptive transfer of DCs loaded with a range of peptide concentrations have likewise suggested that low TCR signal strength favors Th2 cell induction (59), *in vivo* models of infection or immunization have not reproduced these findings, as many studies have shown that high doses of antigen enhance the generation of Th2 cells (60–68) and that TCR affinity does not affect IL-4 production by activated T cells (69). Therefore, current evidence does not clearly indicate whether TCR signal intensity controls the induction of Th2 cell responses upon *in vivo* exposure to Th2-inducing stimuli. Other studies have tested whether specific costimulatory signals influence Th2 cell differentiation. Particularly, some studies have provided evidence that *in vitro* generated DCs expressing membrane-bound Notch ligands, particularly Jagged 1 and Jagged 2, gain the capacity to instruct Th2 cell polarization (70, 71). However, other studies have shown that Jagged expression by DCs is insufficient or not required for Th2 cell differentiation (72, 73). Furthermore, *in vivo* studies have shown that expression of Jagged Notch ligands on cDCs is dispensable for induction of Th2 cell responses to natural allergens and subsequent allergic airway inflammation (74). Thus, current evidence does not support a role for Jagged expression on cDCs in directing Th2 cell differentiation to allergens. Instead, Notch has been found to orchestrate multiple Th cell programs, including Th1, Th2, Th17, Treg, and Tfh, and has been suggested to be a general and unbiased amplifier of Th cell responses (75).

Importantly, however, initial Th2 cell lineage commitment has been shown to require IL-2 signaling and IL-2-induced activation of signal transducer and activation of transcription 5 (STAT5) (76, 77). Specifically, IL-2-STAT5 signaling is needed to promote transcription of the *Il4ra* gene, leading to increased cell surface expression of *IL-4Rα* (also known as CD124) and subsequently increased responsiveness to IL-4 (77). In addition, IL-2 stabilizes the accessibility of the *Il4* locus, allowing for early IL-4 production (76–78). Thus, IL-2-induced signaling during early Th2 cell differentiation is required to support increased IL-4 production and increased IL-4 responsiveness, allowing for an IL-4-positive feedback amplification loop that preserves the Th2 cell phenotype. cDCs are not a critical source of IL-2. And although group 3 ILCs are a dominant source of IL-2 in peripheral tissues (79, 80), in the secondary lymphoid organs, where T cell priming occurs, IL-2 is thought to be produced primarily by CD4^+^ T cells shortly after their activation by cDCs. IL-2 signaling is first required for the initial clonal T-cell expansion (81, 82). However, IL-2-deficient T cells also show defective Th2 cell differentiation *in vitro* (76, 77), supporting that IL-2 produced by activated T cells is necessary and sufficient to initiate the Th2 cell differentiation program. Remarkably, *in vitro* IL-2 production and responsiveness are favored by weaker TCR signaling (55, 83), which may be one mechanism to explain how TCR signal intensity can control *in vitro* Th2 cell differentiation. However, IL-2 is not exclusively produced during Th2 cell differentiation. Therefore, Th2 cell commitment must be regulated *in vivo* by additional mechanisms. One possibility is that CD4^+^ T cells default into the Th2 cell pathway in the presence of strong IL-2 signaling but the absence of positive signals (or cytokines) that drive differentiation into other Th lineages, particularly into Th1 cells (31–33, 84, 85). The regulatory roles of Th1-inducing signals in inhibiting Th2 cell differentiation will be discussed in detail below.

Accumulating evidence has shown that interactions between naïve CD4 T cells and antigen-presenting cDCs can occur within distinct sub-anatomic regions of secondary lymphoid tissues, leading to the differentiation of specific Th subsets (86, 87). These data suggest that T-cell lineage commitment and the acquisition of district cytokine profile may be controlled by particular localization within secondary lymphoid organs. In particular, Th1-directing stimulations induce cDCs to localize into the deep T cell area of the lymph node *via* a CC-chemokine receptor 7 (CCR7)-dependent mechanism (88–90). This positioning of cDC allows for efficient initial priming of Th1 cell responses (88). On the contrary, during Th2-directing stimulations, antigen-bearing cDCs are preferentially attracted to the border of B cell follicles and T cell zone (T-B border) and to the area between B cell follicles (perifollicular region) rather than to the T cell zone (18, 86, 89, 91, 92). The encounter of cDCs and responding CD4^+^ T cells, specifically within the T-B/perifollicular zone of the lymph node, was found necessary for the establishment of Th2 cell responses, as repositioning of cDCs or T cells to the T cell area compromised Th2 cell priming (86, 89). Thus, this evidence suggests that the T cell area of the lymph node is a specialized microenvironment that contributes to the effective priming of Th1 cells but also is an unfavorable environment for the priming of Th2 cell responses. In contrast, Th2 cell responses are efficiently primed in the T-B/perifollicular region of the lymph nodes. Since the signals that induce Th1 and Th2 cells have antagonistic effects on each other, it is understandable that the differentiation of these two subsets occurs in anatomically separate settings. However, it is more than possible that these particular lymph node microenvironments optimize the differentiation of individual T cell responses. As an example, chemokine signals expressed in the T area that induce the positioning of cDCs within this location also enhance the ability of the cDCs to produce IL-12 and differentiate Th1 cells (93). Besides, T cell priming in the T cell area allows for interaction with blood-recruited monocytes and monocyte-derived DCs (moDCs) (94–96), providing an additional source of IL-12 for optimal Th1 cell differentiation (88, 97–99). On the other hand, Th2 cell differentiation requires strong and sustained IL-2 signaling, which is perhaps best achieved in the T-B/perifollicular region. For example, IL-2 could be more available in the T-B/perifollicular area due to the low presence of Treg cells that consume IL-2 (100). However, questions remain as to how the cDCs integrate signals provided in the environment after Th2-directing stimulations to migrate and be specifically retained in the T-B/perifollicular areas of secondary lymphoid tissues, to provide signals that efficiently support autocrine IL-2 production and signaling in T cells, without providing cytokines that inhibit the Th2 cell differentiation program.

Innate sensing of microbial pathogens or derived products is principally mediated by pattern recognition receptors (PRRs), which recognize molecules frequently found in pathogens (the so-called pathogen associated molecular patterns or PAMPs). PRRs include Toll-like receptors (TLRs), RIG-I-like receptors (RLRs), NOD-like receptors (NLRs), C-type lectin receptors (CLRs), and cytosolic DNA sensors (101). Upon ligand engagement, PRRs trigger intracellular signal transduction pathways, which ultimately result in the expression of a variety of pro-inflammatory and polarizing cytokines and up-regulation of co-stimulatory molecules. These gene products orchestrate the early host response to infection and also are a prerequisite for the subsequent activation and shaping of adaptive immunity. Particularly, pathogen recognition by PRRs and subsequent expression of polarizing cytokines is essential for initiating Th1 and Th17 responses. In contrast, Th2 cell responses can develop or even get enhanced in the absence of PRR-mediated signaling (102–108). Indeed, as it will be discussed in below sections, the trigger of PRR-mediated signaling and subsequent induction of pro-inflammatory cytokine response usually suppresses Th2 cell priming. This is in line with the idea that no positive signal (or cytokine) controls Th2 polarization; instead, it is the absence of one that causes Th2 cell responses to develop. However, the activation of certain PRR in specific cell types or by certain PAMPs can elicit attenuated rather than promote inflammatory cytokine responses and consequently have been shown to act as adjuvants that favor or enhance biased type 2-immunity. For example, TLR2 stimulation promotes weak IL-12 production (109–113), and thus TLR2-mediated signals preferentially stimulate Th2 cell polarization (111, 113, 114). Likewise, polysaccharides commonly found in allergens can bind CLRs such as Dectin-1/CLEC7A and Dectin-2 (115–117). Dectin-1 and Dectin-2 ligands usually prompt attenuated IL-12 responses (118–120), but still can act as adjuvants that promote cDC activation and migration for the subsequent priming of allergen-specific Th2 cells (121–125). But among all PRRs, TLR4 is probably the most ambiguous regulator in the context of type 2-immunity, as its activation can lead to suppression of Th2 cell differentiation or, conversely, be a driving force of Th2 bias. How TLR4 activation can cause these two opposite outcomes in the context of type 2-immunity is still a topic of research. In general, strong stimulation of the TLR4-driven pro-inflammatory response is associated with IL-12 release and suppression of Th2 cell priming (103, 126–128). However, several factors can influence TLR4-mediated signaling and cause an attenuated pro-inflammatory cytokine response that promotes type 2-inflammation. LPS is the best-known and most potent TLR4 agonist and is, therefore, a powerful inducer of pro-inflammatory cytokines (129). But it should be noted that in this context, host sensitivity to LPS is critical when it comes to eliciting a robust pro-inflammatory cytokine response leading to IL-12-producing cDCs and suppression of Th2 cell responses, particularly in response to low amounts of LPS. And therefore, suboptimal responses to low amounts of LPS may promote rather than prevent Th2 responses, thereby favoring allergic airway inflammation (103, 130–132). As will be discussed below, moDCs are the primary sensor cells for low-dose LPS and a key source of pro-inflammatory cytokines. Thus, factors influencing moDC differentiation and activation can switch LPS activity from protective to pro-pathogenic in the context of Th2 cell allergic responses. In this regard, activation of TLR4 in the absence of moDCs initiates, rather than prevents, Th2 allergic inflammation in the lungs (103). When moDCs are absent, LPS primarily drives TLR4 activation in the stroma/epithelium (103, 130, 131, 133). And this context favor Th2 cell development since the TLR4-driven response in structural cells has a low pro-inflammatory component but induces cytokines or “alarmins” that condition cDCs to initiate Th2 cell responses (134). On the other hand, although TLR4 primarily recognizes and is activated by LPS, alternative agonists for TLR4 have also been described (135). Importantly, TLR4 signaling induced by some of these alternative agonists has been shown to promote, rather than suppress, type 2-driven inflammation (136–139). A common feature of these TLR4 agonist ligands is that they induce alternative activation of TLR4, resulting in weakened inflammatory response. Canonical activation of TLR4 by LPS is absolutely dependent on the presence of myeloid differentiation factor-2 (MD-2), which binds LPS and initiates TLR4 pro-inflammatory cytokine signaling (140–142). However, alternative TLR4 ligands that elicit type 2-immunity activate TLR4 in the absence of MD-2 (137–139). The proposed mechanism for this alternative/non-canonical TLR4 activation is that these ligands are proteins with structural and functional homology to MD-2 that can reconstruct TLR4 signaling in the absence of MD-2 (137). Future studies will be required to understand precisely how canonical and non-canonical TLR4 activation pathways can elicit different cytokine profiles and thus enable distinct TLR4 functions.

**Figure 1:**
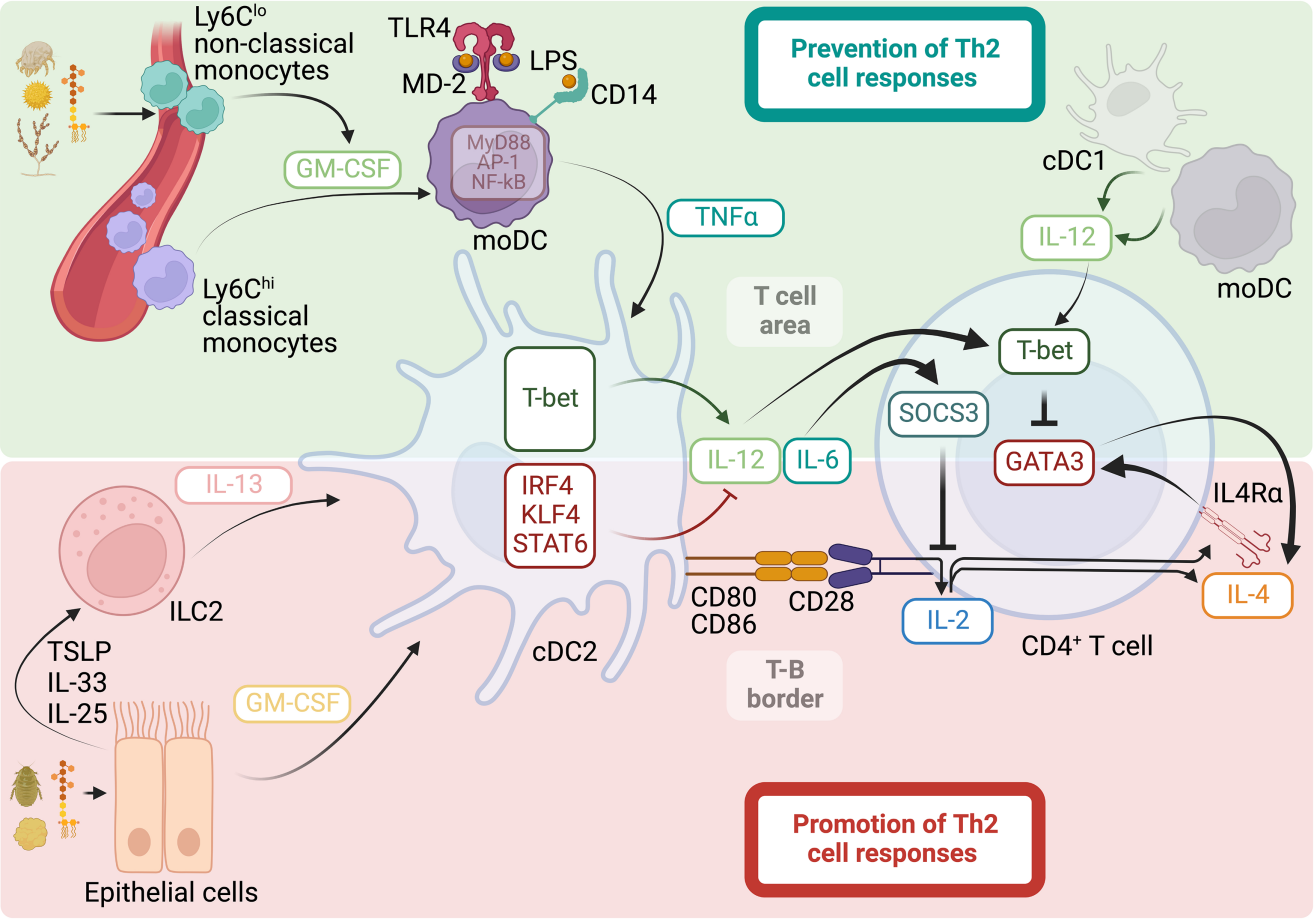
Mechanisms that promote and prevent Th2 cell responses to allergens. Promotion of Th2 cell responses: allergens with proteolytic activity, cytokines, and microbial products, such as LPS, can activate epithelial cells for subsequent release of the cytokines GM-CSF, TSLP, IL-33, and IL-25. This leads to the activation of ILC2s and the release of IL-13. GM-CSF and IL-13 stimulate the migration and expression of the transcription factors IRF4, KLF4, and STAT6 in cDC2s, ultimately promoting the functional specialization of the cDC2s to support Th2 cell differentiation by reducing the ability of the cDC2s to produce IL-12, while retaining their co-stimulatory ability to foster strong IL-2 responses in CD4^+^ T cells. Strong and sustained IL-2 signaling in the absence of IL-12 promotes Th2 cell lineage commitment by promoting the expression of IL-4Rα and IL-4, allowing for an IL-4-positive feedback loop that initiates and preserves the Th2 cell phenotype. Prevention of Th2 cell responses: allergens with cysteine protease activity stimulate GM-CSF release from perivascular Ly6C^lo^ non-classical monocytes, guiding the differentiation of Ly6C^hi^ classical monocytes into moDCs. GM-CSF licenses an inflammatory signature in moDCs by increasing the expression of TLR4, CD14, and intracellular signaling members involved in the MyD88/NF-kB/AP-1-dependent pathway. Functional programming of moDCs by GM-CSF allows these cells to increase their sensitivity to LPS and stimulate the production of the pro-inflammatory cytokine TNFα, which guides cDC2 activation for Th2 cell suppression rather than promotion. In particular, TNFα induces the expression of the transcription factor T-bet in cDC2s, which is intrinsically necessary for the ability of cDC2s to produce sustained IL-12 and suppress Th2 cell priming by inducing T-bet and inhibiting GATA3 in CD4^+^ T cells. cDC2s can also produce IL-6, upregulating SOCS3 in CD4^+^ T cells and ultimately suppressing IL-2 signaling and early Th2 cell commitment. cDC1s and moDCs are additional sources of IL-12 that contribute to the suppression of Th2 cell responses.

In addition to their ability to activate PRRs, allergens with strong ability to elicit Th2 cell responses, such as HDM, pollen, fungi, or cockroaches, share the common feature of containing proteases (143). Moreover, proteolytic enzymes extracted from these allergens have the ability to trigger potent Th2 cell responses (116, 144–146). Thus, the presence of protease activity in allergens is thought to play an important role in initiating Th2 cell responses. Additionally, several cell populations and mediators have been shown to support cDC activation and migration and, thus, have been found to be central regulators of Th2 cell immunity. Principally, activation of epithelial cells by microbial products, cytokines, or allergens with proteolytic activity and the consequent release of the “alarmin” cytokines thymic stromal lymphopoietin (TSLP), IL-33, and IL-25 is thought to be the initial step for the subsequent activation of cDCs and priming of allergen-specific Th2 cells (134, 147, 148). In support, alarmin cytokines and their receptors are major susceptibility loci for human asthma (149–151). Importantly, TSLP, IL-33 and IL-25 are potent activators of ILC2, which produce IL-13 in response to these stimulations (53, 152–158). IL-13 production by ILC2 has been shown to be important for the initiation of Th2 cell responses after allergic sensitization or immunization through the skin or the respiratory tract (53, 152). Mechanistically, IL-13 produced by ILC2 has been shown to promote the migration of cDCs into the draining lymph node and their functional specialization to stimulate naïve T cells to differentiate into Th2 cells (53, 152). cDCs can be divided into two ontogenically distinct subsets, the type-1 cDCs (cDC1s) and type-2 cDCs (cDC2s) (159). In the context of induction of allergen-specific CD4^+^ T-cell responses, cDC2s are especially adept at capturing and transporting allergen-derived proteins from the tissue into the draining lymph node and presenting them to CD4^+^ T-cell to induce their activation and polarization (18, 46, 103, 128). The integration of the signals in the environment defines the functional specialization of cDC2s and ultimately determines their ability to either promote (18, 46–51, 53) or prevent (103, 128) Th2 cell responses to allergens. The specific signals driving cDC2 diversification are gradually being characterized. The expression of the transcription factors Interferon regulatory factor 4 (IRF4) (48, 50), Krüppel-like factor 4 (KLF4) (51), and STAT6 (53) promote the functional specialization of cDC2s to favor Th2 cell differentiation. In contrast, and as will be discussed below, the induced expression of the transcription factor T-bet is intrinsically necessary for the ability of cDC2s to produce sustained IL-12 and suppress Th2 cell priming (103, 128). Thus, these data suggest transcription factor-guided functional specialization of cDC2s and the influence of environment-derived signals in driving this process. Indeed, IRF4 controls unresponsiveness to TLR4 stimulation in cDC2s and, therefore, imprints a low capacity to produce IL-12 in response to LPS (48, 160–162). Additionally, KLF4 and STAT6 enable cDC2s to respond to IL-13, thus guiding the function of cDC2s to promote Th2 cell differentiation, most likely by similarly reducing the cDC2 ability to produce IL-12 in response to PAMPs, while preserving their CD4^+^ T cell stimulatory capacity (53, 163, 164). Overall, the data suggest that the ability of cDC2 to suppress IL-12 production and the prevention of naïve CD4^+^ T cells from receiving IL-12 signaling are decisive for inducing Th2 cell responses. Furthermore, the data suggest that this can be controlled by the transcriptional programming of cDC2s and by particular location of DC-T cell interactions away from the T cell areas of secondary lymphoid tissues (Figure 1).

## Generation of effector Th2 cell responses in the lung

Experimental mouse models of allergic response to inhaled allergens have been instrumental in investigating the mechanisms underlying the initiation and maintenance of allergen-specific Th2 cell responses. Using these models, it has been shown that the development of allergic Th2 cell responses through the intranasal (i.n.) route usually occurs in two steps. The first step is called “*sensitization*” and is initiated after the primary i.n. exposure to an allergen. During the initial sensitization lung-migratory cDC2s traffic into the lung-draining lymph nodes to prime allergen-specific CD4^+^ T cells with a type 2/Th2-biased cytokine profile (15, 18, 46, 47, 50, 51). Importantly, however, this initial exposure does not typically result in the accumulation of effector allergen-specific Th2 cells in the airways (15, 18) and therefore does not necessarily cause clinical manifestations. Instead, allergen sensitization triggers a strongly biased Tfh2 cell response that is restricted to the lung-draining lymph nodes (15, 18, 165). The second step is characterized by the development of pathology and clinical features following secondary or subsequent allergen exposures or “c*hallenges*.” This phase is characterized by the accumulation of effector allergen-specific Th2 cells in the airways and Th2 cytokine production (15, 18).

**Figure 2:**
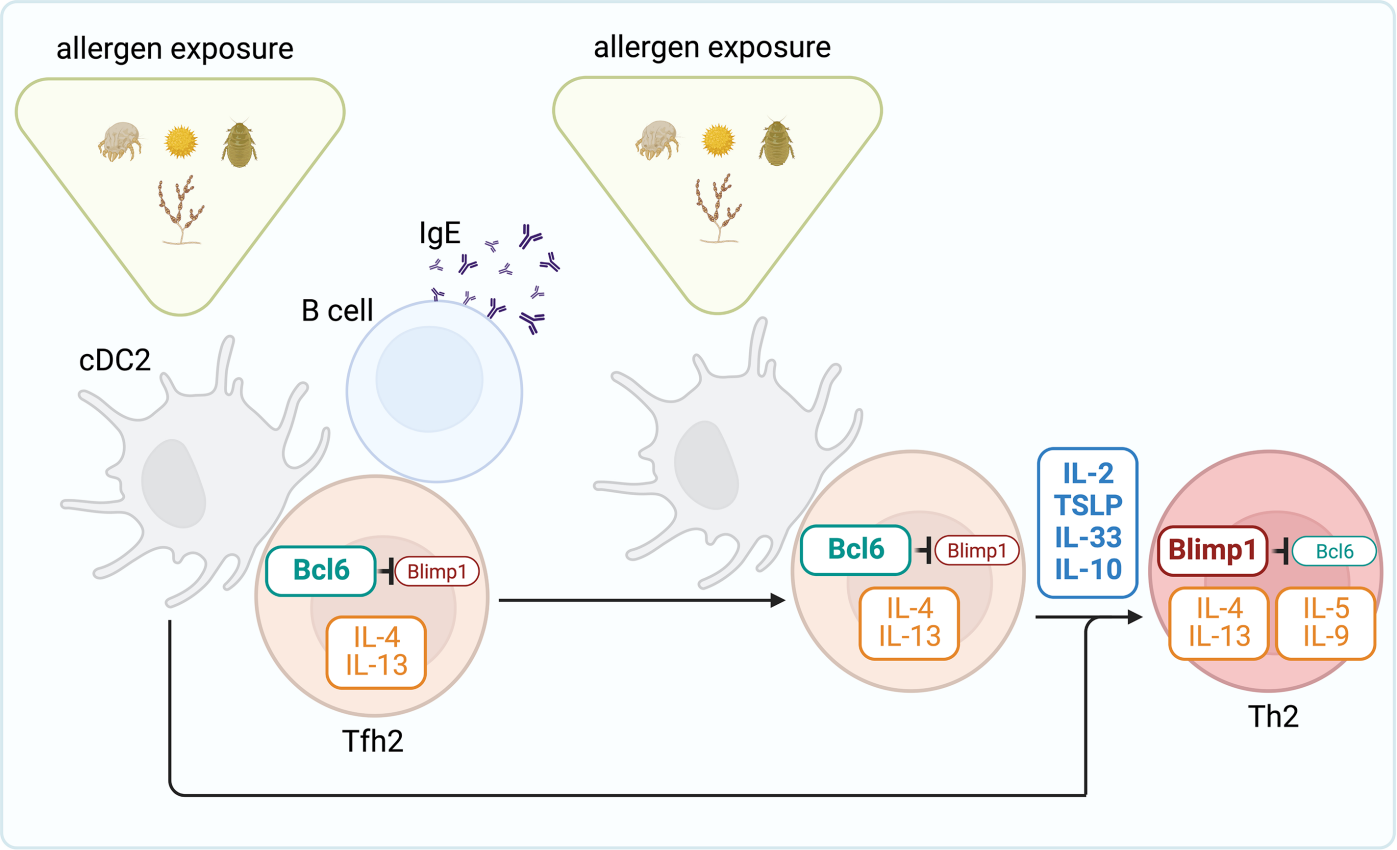
Steps toward generating effector Th2 cell responses to allergens. During the sensitization phase, cDC2s prime allergen-specific Tfh2 cell responses in the draining lymph node. Through interactions with B cells, Tfh2 cells promote IgE secretion. Following allergen re-exposure, Tfh2 cells differentiate into effector Th2 cells that subsequently migrate to the lung and promote allergic airway inflammation. Tfh2 cell development depends on the expression of the transcription factor Bcl6, which functions as a transcriptional repressor that prevents Blimp1 expression and the acquisition of the Th2 effector program. The cytokines IL-2, TSLP, IL-33, and IL-10 promote Blimp1 expression and thus drive differentiation of Th2 effectors from tfh2 cells.

Tfh2 cells can produce large amounts of Th2 cytokines, including IL-4 and IL-13, in response to allergens and helminths (12, 18, 89, 166–170) and, in fact, are the primary sources of IL-4 and IL-13 during the sensitization phase (12, 18). IL-4/IL-13-producing Tfh2 cells are critical for the sustained production of IgG1 and IgE class switching (166, 167, 169) and high-affinity IgE (12). A division of labor between IL-4-producing Tfh2 and IL-13-producing Tfh2 [also known as Tfh13 (12)] has been proposed, where IL-4-producing Tfh2 instruct the production of IgG1 and low-affinity IgE, but IL-13 production drives high-affinity IgE secretion and anaphylaxis (12). In animal models, IL-13-producing Tfh2 are not induced after infection by helminths (12, 170), but they can be generated to aeroallergens, including HDM and fungal allergens in some studies (12), but not in others (171). But, besides controlling B cell isotype switching, Tfh2 cells generated during the sensitization phase can survive in the lymph nodes as memory cells and have the unique ability to give rise to effector Th2 cells upon allergen rechallenge (18). These data suggest lineage flexibility of Tfh2 cells in allergic disease and identify these cells as a crucial reservoir of Th2 cell progenitors. Tfh cell development depends on the expression of the transcription factor B cell lymphoma 6 (Bcl6), which functions as a transcriptional repressor that prevents the acquisition of T effector programs (172). In contrast, the generation of effector Th2 cells in the lung requires expression of the transcription factor B-lymphocyte-induced maturation protein 1 (Blimp1), encoded by the Prdm1 gene (173). Bcl6 and Blimp1 are reciprocally antagonistic transcription factors (174, 175). Thus, the balance between Blimp1 and Bcl6 expression likely controls the relative commitment of CD4 T cells to the effector Th2 or Tfh2 cell pathways. Taken together, the two-step model for the development of allergic Th2 cell responses predicts that induced expression of Bcl6 in T cells (favored during sensitization to inhalant allergens) drives Tfh2 cell differentiation and central memory. In contrast, induced expression of Blimp1 during allergen re-exposure promotes effector Th2 cells that migrate to the lungs (Figure 2). Consistent with this model, upregulation of Bcl6 expression upon allergen exposure inhibits effector Th2 cell fate choice (171), whereas the up-regulation of Blimp1 favors the differentiation of effector Th2 cells at the expense of Tfh cells (173). Understanding what signals control Bcl6 and Blimp expression in allergen-specific T cells will be essential for understanding Tfh vs. effector fate choice. Like in other immune responses (172), the acquisition of Bcl6 by allergen-specific T cells is initiated by cDC priming but then maintained by cognate interactions with B cells (15, 18). Cognate B cells preserve the pool of allergen-specific Tfh2 and provide a niche for memory development and maintenance (18). In contrast, the loss of B cell interactions favors the effector Th2 cell pathway (171). Thus, the crosstalk between T cells and B cells likely regulates the balance between Tfh and effector Th2 cell differentiation. On the other hand, Blimp1 expression increases in response to IL-2 acting through STAT5 (176, 177), which has been shown to be a potent inhibitory mechanism of Bcl6 and Tfh cell responses (178, 179); but it enhances the generation of effector Th2 cells migrating to the lung (171). Similarly, TSLP activates STAT5, which represses Bcl6 and stimulates effector pathogenic Th2 cell responses (180). IL-10 can also upregulate Blimp1 *via* STAT3 (181) and has been shown to promote effector Th2 responses and the development of allergic lung disease by repressing Bcl6 expression (173). Finally, IL-33 can likewise promote Blimp1 expression in T cells and exacerbate allergic airway inflammation (182) (Figure 2). Future studies will need to determine the factors that control the production of these cytokines, their source, and their relative contribution to the effector Th2 and Tfh2 cell pathways. Notably, the antagonism between Bcl6 and Blimp1 has been shown to control the balance of Tfh2 vs. effector Th2 pathways during respiratory but not cutaneous allergen sensitization (173, 183). Moreover, the nature of the allergen can also affect the differential development of Tfh2 and effector Th2 cells (166). Thus, it will also be necessary to address how different mechanisms govern during sensitization with diverse natural allergens and different routes.

## Mechanisms that suppress Th2 cell priming by dendritic cells

T-bet is a critical transcription factor for Th1 cell differentiation. T cells lacking T-bet fail to develop into Th1 cells (85). But, loss of T-bet also results in the activation of the Th2 cell differentiation program (33, 85, 184, 185). Moreover, overexpression of T-bet in Th2 cells results in the loss of the Th2 cell phenotype (85, 185). Thus, T-bet plays a crucial role in suppressing the Th2 cell-associated program. GATA Binding Protein 3 (GATA3) is the critical transcription factor for Th2 cell differentiation (186–190). IL-4-mediated STAT6 activation is the main inducing signal for GATA3 upregulation and maintenance (188, 191). Mechanistically, T-bet suppresses the Th2 cell differentiation program by directly inhibiting GATA3 expression through epigenetic repression at the *Gata3* locus (33, 185) and inhibiting GATA3 function through protein-protein interactions (192).

*In vitro* and *in vivo* animal models have essentially contributed to the understanding of the role of T-bet in preventing Th2 cell development. However, an important question is to demonstrate whether T-bet plays a significant role in actively preventing allergen-specific Th2 cell development in healthy non-atopic individuals. Recently, the first patient with autosomal recessive complete T-bet deficiency has been reported (193–195). This human T-bet deficiency causes disrupting development of IFNγ-producing cells (193). But importantly, the patient also developed upper airway inflammation, peripheral eosinophilia, and increased production of the Th2 cytokines IL-4, IL-5, and IL-13 due to Th2 skewing of T-bet-deficient CD4^+^ T cells (194). Additionally, a B cell class switching to IgG1, IgG4, and IgE has been observed in this patient (195), possibly due to the favored induction of Tfh2 and Th2 cell cytokine production (194). Thus, T-bet contributes to preventing Th2-biased differentiation in humans. Interestingly, T-bet protein is downregulated in T cells from the airways of human patients with Th2-driven asthma (184). More recent human studies have further found that activated allergen-specific T cells exist in both healthy and allergic/asthmatic subjects (196). However, the polarization profile of the reactive T cells was different. Allergic/asthmatic had allergen-specific T cells with a Th2 cell profile, whereas allergen-responsive T cells in healthy/non-allergic subjects were characterized by a Th1/IFN signature (196). Overall, these data suggest that T-bet plays an essential role in preventing Th2 cell priming and Th2 cell-driven allergic airway disease in humans.

Allergens contain endogenous PRR agonists or can be contaminated with exogenous environmental PRR ligands. Several studies have indicated that exposure to allergens with high content of microbial products, particularly bacterial endotoxins or LPS, inversely correlates with the development of allergen-induced, Th2-driven diseases such as allergic asthma and atopy (22–30). This data suggests that LPS detection is linked to the suppression of Th2 cell immune responses to allergens. As a note, among all the pro-inflammatory PRR ligands that can be found in allergens, it is not surprising that LPS shows the most robust correlations due to its extreme potency. But, since TLRs have redundant functions, various TLR ligands could likely suppress th2 responses through similar mechanisms. Using animal models, we have demonstrated that sensitization with HDM allergens containing low amounts of LPS can very efficiently prevent Th2 cell-driven allergic inflammation, particularly in adult mice (128). LPS prevents Th2-dependent allergic responses by enabling IL-12 production by lung-migratory cDC2s. Consequently, allergen-specific T cells interacting with IL-12-expressing cDC2s can upregulate T-bet (128), which precludes the Th2 cell differentiation program and subsequent pathogenic allergic response to HDM allergens. T-bet can be redundantly upregulated by IL-12 and IFNγ (33, 197–199). IFNγ produced by T cells induces T-bet expression, which serves as a positive feedback loop that allows complete Th1 cell differentiation (198, 200). But during initial priming, it is widely accepted that IL-12, produced primarily by cDCs, initiates Th1 cell differentiation by promoting the early upregulation of T-bet. T-bet then stimulates IFNγ production, allowing for the positive feedback loop that maximizes Th1 immunity when IL-12 becomes limited (31, 33, 201, 202). Besides controlling Th1 cell differentiation, T-bet suppresses the Th2 cell differentiation program to aeroallergens, where IL-12 produced by cDC2s plays a crucial role in the process (128). Importantly, sensitization with allergens containing low amounts of LPS induces moderate levels of T-bet in allergen-responsive T cells. Although these moderate levels of T-bet expression are sufficient to inhibit Th2 cell differentiation, they do not cause substantial IFNγ production or Th1 cell differentiation (128). Thus, different levels of T-bet expression may differentially control Th2 cell suppression, Th1 cell generation, and the acquisition of effector functions (203).

Migratory cDC2s can either promote (18, 46–51, 53) or suppress (103, 128) Th2 cell responses to allergens, and in this regard, the activation state of cDC2s is crucial to define their role. As previously discussed, the transcription factors IRF4, KLF4, and STAT6 program cDC2s to support Th2 cell differentiation, most likely by reducing the ability of cDC2s to produce IL-12 (48, 50, 51, 53, 160–162). We have found, otherwise, that the transcription factor T-bet promotes the functional specialization of cDC2s to suppress Th2 cell differentiation by promoting the ability of cDC2s to produce sustained IL-12 after sensitization with allergens containing low amounts of LPS (103, 128). Therefore, the ability of cDC2s to promote or prevent Th2 cell responses is highly dependent on their acquired ability to express T-bet and produce IL-12 (Figure 1). Although the ability of cDC2s to produce IL-12 appears to be tightly regulated and correlates with its functional ability, cDC1s constitutively express high levels of IL-12 (128, 204, 205). As such, cDC1s are commonly associated with the Th1-inducing cDC subset (159). Additionally, cDC1s have been shown to contribute to ameliorating Th2 cell responses to allergens (206, 207). However, cDC2s can also produce large amounts of IL-12 when properly stimulated (97, 208–210). Furthermore, IL-12-producing DC2s are the main contributors to the prevention of Th2 cell responses upon host detection of low levels of LPS in aeroallergens (103, 128). As such, cDC2s can normally prevent Th2 cell differentiation in mice deficient in Basic leucine zipper ATF-like transcription factor 3 (BATF3) despite the absence of cDC1s; but they fail to suppress allergic Th2 cell responses when they cannot express T-bet and produce IL-12 (128). In conclusion, cDC2s can activate either a pro- or anti-Th2 cell phenotype (Figure 1). Future studies should determine the full picture by integrating the heterogeneity of transcriptional programs that underlie functional specification in cDC2s.

As mentioned, host recognition of LPS and robust induction of pro-inflammatory cytokine responses are linked to the suppression of Th2 cell immune responses to allergens. Thus, in this context, factors that influence or modify LPS responsiveness have the potential to alter Th2-driven allergen sensitization. LPS can be detected by multiple cells expressing TLR4. Predominantly, myeloid cells such as neutrophils, macrophages, cDCs, and inflammatory monocytes can very efficiently respond to LPS since they express optimal levels of TLR4 and the co-receptors CD14 and MD-2. Among myeloid cells, classical monocytes express the highest levels of these proteins (211). CD14 binds LPS and transfers the bound LPS to the TLR4-MD-2 complex (212). This transfer cascade is essential for the efficient triggering of the TLR4-induced inflammatory cytokine response involving activation of the TIRAP-MyD88 and TRAM-TRIF signaling pathways (141, 213, 214). Non-hematopoietic cells such as vascular (215), epithelial (216), and adipose cells (217) can also express TLR4. However, unlike myeloid innate immune cells, these structural cells express low levels of CD14 and MD-2 and have been shown to be poorly responsive to LPS under normal conditions (218–221). Given the diversity of cells that can respond to LPS, this can lead to highly variable sensitivities to LPS and qualitatively and quantitatively very different cytokine responses depending on the cell type(s) involved. In this regard, as discussed previously, it has been shown that LPS-driven TLR4 signaling in stromal cells, most likely in airway epithelial cells, may favor the initiation of airway Th2 cell responses to proteins that would otherwise behave as inert antigens (103, 130–132, 222). Stromal or epithelial cell-guided LPS responses have a low inflammatory component; however, they could still stimulate the production of proallergic cytokines or “alarmins” and the consequent activation and migration of antigen-containing cDCs (15). Conversely, LPS-induced TLR4 activation in hematopoietic cells is needed to suppress Th2 cell sensitization to natural allergens (103). However, the inhibitory functions of cDC2s on Th2 cell allergic responses do not require direct recognition of LPS (103). Instead, this function in cDC2s is controlled by inflammatory mediators produced by classical Ly6C^hi^ monocytes that activate the differentiation program into moDCs (103). Granulocyte-macrophage colony-stimulating factor (GM-CSF) is critical for moDC differentiation (103, 223, 224), and GM-CSF-driven functional programming of moDCs allows these cells to increase their sensitivity to LPS and their ability to elicit a pro-inflammatory cytokine response (103, 225–228). In particular, GM-CSF licenses the inflammatory signature in moDC by increasing the expression of TLR4, CD14, and intracellular signaling members involved in the TIRAP-MyD88-dependent pathway (103, 229). In contrast, the TRIF-TRAM-dependent pathway appears to be downregulated by GM-CSF signaling in classical monocytes (103). The TIRAP-MyD88-dependent pathway induces rapid activation of nuclear factor κB (NF-kB) and activator protein 1 (AP-1), ultimately leading to the production of inflammatory cytokines such as tumor necrosis factor-α (TNFα) (230, 231). The TRIF-TRAM-dependent pathway is otherwise responsible for mediating type-1 IFN production and signaling (232, 233). Thus, GM-CSF preferentially licenses for the production of TNFα and other pro-inflammatory cytokines in classical Ly6C^hi^ monocytes while inhibiting their type 1 IFN response. Overall, the functional programming of moDCs by GM-CSF allows these cells to increase their sensitivity to LPS and stimulate the production of pro-inflammatory cytokines that allow indirect activation of lung-migratory cDC2s for Th2 cell suppression rather than promotion (Figure 1). Therefore, GM-CSF-driven moDCs have an essential role as amplifiers and modifiers of LPS functions in allergic inflammation.

GM-CSF can be produced by multiple cells types, including hematopoietic (e.g., T cells, macrophages, monocytes, mast cells) and non-hematopoietic (e.g., vascular endothelial cells, epithelial cells, and fibroblasts) cells (234). GM-CSF is produced locally, where it regulates cell function in close proximity. Homeostatic GM-CSF production is required for the differentiation of alveolar macrophages (235, 236). But, in general, GM-CSF production is usually linked to infection, inflammation, or pathological conditions. As such, a number of pro-inflammatory mediators can induce the expression of GM-CSF (237, 238). Allergens can also induce GM-CSF production by different cell types (103, 133, 239). Allergens with cysteine protease activity have a strong capacity to trigger robust GM-CSF response and promote moDC differentiation. In particular, cysteine protease activity on allergens prompts GM-CSF production by perivascular non-classical Ly6C^lo^ monocytes, thereby guiding the differentiation of classical Ly6C^hi^ monocyte into moDCs (103). Conversely, trypsin-like serine protease activity or the presence of endogenous TLR4 ligands on allergens activate lung epithelial cells to induce GM-CSF production (133, 240). Importantly, the production of GM-CSF by these two different cellular compartments, which release GM-CSF at different locations and in different amounts, appears to have completely opposite results in allergen sensitization. Epithelial-derived GM-CSF has been shown to play a critical role in allergic sensitization, as this source of GM-CSF promotes the activation and migration of cDCs from the lung to the lymph node, where they prime Th2 cell responses (103, 133, 240–243). In contrast, production of GM-CSF in perivascular areas by non-classical monocytes regulates the *de novo* generation of moDCs from newly-recruited classical monocytes, which ultimately instigates cDC2s for Th2 cell suppression to LPS-contaminated allergens (103) (Figure 1). Although classical Ly6C^hi^ monocytes are critical mediators of inflammatory responses, non-classical Ly6C^lo^ monocytes have been widely viewed as anti-inflammatory, as they maintain vascular homeostasis and promote reparative processes (244–248). However, non-classical monocytes are also a first line in the recognition of immunological insults and can contribute to inflammation (249–254). Future research should determine whether nonclassical monocytes could control the inflammatory functions of classical monocytes in settings other than allergen exposure and cysteine protease-mediated activation. Overall, the published mechanistic data suggest the interplay between the GM-CSF response elicited after allergen exposure by different cellular sources and the amount of endotoxin/microbial products contained in those inhaled allergens can oppositely balance the activation of mDC2s and, in the end, establish whether the stimulated allergen-specific T cell response will be protective or pathogenic. In view of this, an altered response to TLR ligands or GM-CSF conditioned by polymorphisms that affect the function of these signaling pathways might be associated with altered susceptibility to allergen sensitization. Many studies have tested this hypothesis and have found an association between TLR or GM-CSF polymorphisms and sensitization to airborne environmental allergens and allergy development. For example, single nucleotide polymorphisms (SNPs) in CD14, TLR4, and TLR2 genes have been correlated with the development of allergy sensitization and symptoms of allergy in children (255–264). Likewise, variants in the gene for GM-CSF have been associated with the development of atopic diseases in children (265–267). In contrast, other studies have shown no association between CD14, TLR2, TLR4, or GM-CSF polymorphisms and allergy (268–272). Thus, although polymorphism in genes encoding GM-CSF and TLR signaling members is associated in many cases with allergic phenotypes, results are variable. In general, stronger associations have been found in children living in environments that expose them to high levels or a different diversity of microbial agents, suggesting that genetic variants on allergy susceptibility appear to be modified by quantitative factors (levels of exposure) and qualitative components (the type of microbial products/TLR ligands present in the environment) (255, 258, 260, 261, 263). Therefore, gene-by-environment interactions between functional gene polymorphisms and environmental exposures should be considered together to better understand susceptibility to allergic sensitization [reviewed in (273)].

Although, as discussed in this section, IL-12 and T-bet play essential roles in the suppression of Th2 cell differentiation, we have recently discovered that IL-6 also plays a crucial and T-bet-independent role in inhibiting the Th2 cell differentiation program [preprint (274)]. Loss-of-function mutations that affect IL-6 signaling, including IL6 receptor (IL-6R) (275), Glycoprotein 130 (GP130) (276, 277), and STAT3 (278–281), lead to increased Th2 bias and manifestations of allergy (282). Moreover, the *IL6R* locus has been associated with allergy (283, 284). Although these studies with patients suggest a significant role for IL-6 in controlling Th2 bias, the specific contribution of IL-6 and the underlying mechanism remain largely undefined. As discussed, Th2 cell lineage commitment requires strong and sustained IL-2 signaling (76, 77). Our data found that IL-6 suppresses IL-2 signaling during early T cell activation, thereby inhibiting Th2 priming [preprint (274)]. IL-6-driven inhibition of IL-2 signaling in allergen-responding T cells is mediated by the upregulation of the suppressor of cytokine signaling 3 (SOCS3). Thus, the IL-12-T-bet and IL-6-SOCS3 axes cooperate to inhibit the Th2 cell differentiation program by inhibiting GATA-3 and IL-2 signaling in allergen-responsive T cells, respectively (Figure 1).

## Mechanisms that favor Th2 biased immune responses at an early age

**Figure 3:**
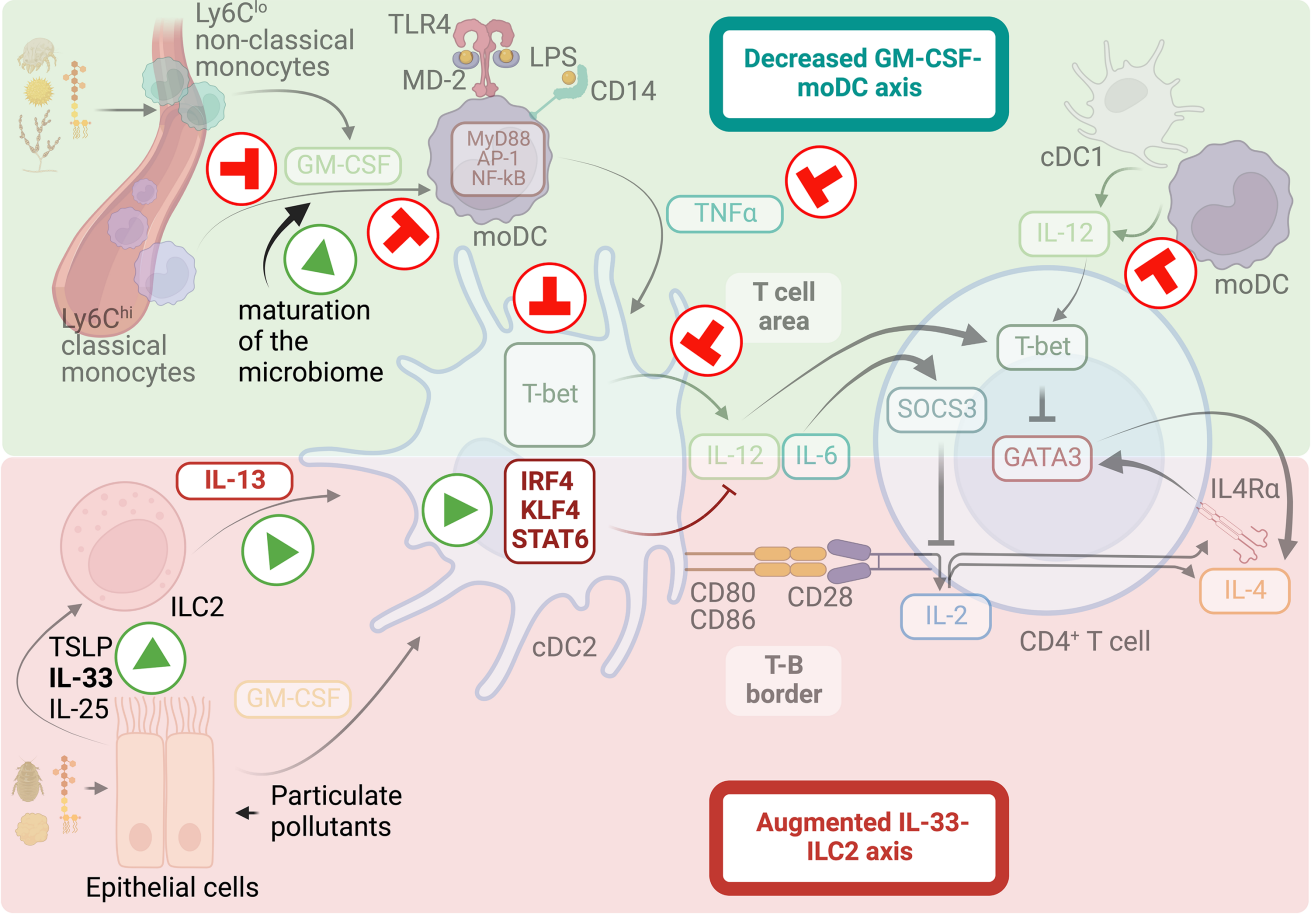
Mechanisms that favor Th2-biased immune responses at early ages. Infants and young children are at higher risk of developing Th2 cell allergic responses than adults. The lack of inhibitory signals and the intensification of triggering factors contribute to the increase in Th2-biased immunity and sensitization to allergens during early childhood. A malfunctioning GM-CSF-moDC axis conditions for LPS hyporesponsiveness during infancy, hindering the induction of T-bet expression on cDC2s and thus driving deficient IL-12 responses. Early dietary practices and exposure to environmental microorganism may shape the maturation of the infant microbiome into compositions that enhance the GM-CSF-moDC axis and protect against sensitization to airborne allergens. On the other hand, a hyperactive IL-33 axis operating in the lung during the early postnatal period increases IL-13 production by ILC2, which activates the migration and pro-Th2 function of cDC2s. Additionally, exposure to inhalable particulate pollutants can intensify this tendency.

It is well known that infants and children are at a higher risk of developing Th2 cell allergic responses than adults. Therefore, they suffer from a high prevalence of respiratory allergic diseases (19, 285). Indeed, observational studies have shown that primary sensitizations to airborne allergens develop gradually and peak in children one to three years of age (7), and are associated with the later development of allergic diseases and asthma, usually before the age of five (5, 6, 8). Importantly, the incidence of childhood allergic rhinitis and asthma has dramatically increased over the past decades in the U.S. and other industrialized nations (6, 10, 286). As such, pediatric asthma is considered the leading chronic disease of childhood. However, the immunological mechanisms underlying the high susceptibility to allergic airway disease in infants and children and the sharply increased burden of allergies and asthma in children in the developed world remain poorly understood. Urbanization is thought to play a major role, as allergic lung diseases are becoming more common in children in urban areas, although the prevalence remains low in rural areas (287–289). Rural/traditional farming settings are associated with close contact with livestock and increased exposure to endotoxin-contaminated aeroallergens in dust (290). Furthermore, epidemiological studies indicate that early exposure to endotoxin-contaminated aeroallergens in those traditional rural/farming settings protects against asthma/allergic reactions in school-age children, whereas infants sensitized to common circulating allergens in low-LPS environments are at increased risk of becoming atopic and developing symptoms of allergic disease later in life (22–30, 291). As discussed previously, laboratory mechanistic studies have established that exposure to airborne allergens containing endotoxin protects against allergic inflammation by preventing the Th2 cell differentiation program in allergen-specific T cells (103, 107, 128). These mechanistic studies have further shown that while low-LPS exposure during allergen sensitization is sufficient to protect adult mice from developing Th2-driven allergic inflammation in the lungs, infant mice require higher doses of LPS (128). These data indicate that LPS prevents Th2-dependent allergic responses with different thresholds in adults and infants, thus providing a plausible mechanism underlying the higher susceptibility to allergic disease and asthma, particularly observed in children living in urban areas and exposed to low-LPS contaminated aeroallergens. As mentioned, mouse studies have further demonstrated that suppression of Th2-driven immune responses to allergens depends on the production of pro-inflammatory cytokines by classical monocytes, particularly TNFα, upon TLR4 engagement (103, 128). However, during infancy, LPS hyporesponsiveness has been observed. In particular, human studies have shown that monocytes from newborns and infants produce significantly fewer pro-inflammatory cytokines, with markedly impaired TNFα release, after stimulation with low concentrations of LPS (292–295), which has been associated with decreased expression of TLR4, CD14, and MyD88 in neonatal monocytes in some studies (293), but not all (296). Corresponding with this, we have also observed that classical monocytes from infant mice produce less TNFα in the lungs after low-LPS sensitization. Furthermore, we have observed that impaired production of TNFα at low doses of LPS is particularly evident in mice during a time window from day 7 to day 20 after birth, after which LPS-driven TNFα production rapidly reaches adult levels (128). Since TNFα released by monocyte-derived cells tunes cDC2 activation to produce IL-12 for subsequent inhibition of Th2 cell sensitization to inhaled allergens (103, 128), it is easy to infer that the inability of infant monocytes to respond to LPS and produce optimal amounts of TNFα is a major cause underlying the increased risk of developing Th2 responses to allergens during infancy (Figure 3). However, how age modulates the ability of classical monocytes to respond to LPS is a topic of further investigation. GM-CSF is a critical factor controlling the expression of CD14, TLR4, and TLR signal transduction proteins in monocytes and, thus, their LPS sensitivity and responsiveness (103). Reduced GM-CSF production and GM-CSF mRNA expression have been observed in blood mononuclear cells from human newborns compared to adults (297–300). Furthermore, studies in mice have shown that innate myeloid cells in the lung respond poorly to GM-CSF (301). These studies suggest that the GM-CSF axis as an amplifier of LPS responses may be functioning poorly at early ages. The cause of these changes during early life is unknown. The microbiome plays an essential role in the maturation of the infant immune response, and indeed, the establishment and development of the gut, lung, and skin microbiota in early life occurs in parallel with the acquisition of immune functions (302–312). Therefore, it is possible that early infant intestinal, lung, and skin exposure to commensal bacteria and fungi may modulate the GM-CSF-TLR4 axis and, thus, LPS responsiveness. Some evidence detailed below supports this hypothesis. Farming-related exposures, which, as discussed above, decrease the risk for allergic outcomes, have been associated with early upregulation of CD14 and TLR expression in the first year of life (313–316). These data suggest that although newborns naturally develop dampened TLR responses, specific environmental exposures during the first year of life contribute to the upregulation of innate immune receptors. Early upregulation of TLR and CD14 expression likely leads to an increased ability to generate inhibitory signals for Th2 cell development and ultimately to reduced risk of allergic diseases. Many recent studies have attempted to identify early life exposures associated with protection against allergies. Children living on farms are exposed to a wider range of environmental bacteria and fungi. The greater diversity of environmental microbial exposure is correlated with protection from allergic airway disease (317). In addition, consumption of raw farm milk (313, 316, 318, 319) and dairy products (320–322) have been identified as exposures that contribute to the protective effect of farm life on childhood allergies. Furthermore, the diversity of the foods introduced in the first two years of life has also been associated with protection from allergic diseases (320, 321, 323). All of these exposures are suggested to play a pivotal role in the evolution and establishment of the microbiome from infancy toward the acquisition of adult-like communities (324–326). Mouse studies have evaluated how specific changes in the composition of the gut and lung microbiota with age, diet, or exposure to environmental microorganisms are associated with protection against allergic diseases (310, 312, 325). In particular, the increase in bacteria from the phylum Bacteroidetes and their ability to ferment fiber in short-chain fatty acids (SCFA) have been shown to modulate the cDC2 and moDC compartments, leading them to a maturation profile that is ineffective in driving Th2 cell responses (310, 325). Thus, metabolites produced by the microbiota can influence the functional specialization of cDC2s and moDCs to prevent Th2 cell responses to allergens. Interestingly, early-life dietary practices associated with protection from allergic disease have also been correlated with the maturation of the gut microbiome and elevated SCFA levels (325, 327, 328). Overall, dietary and environmental microbial exposures shape the maturation of the infant microbiome toward an adult-like structure. The data indicate that early maturation of the infant microbiome helps prevent the development of Th2 cell responses, thus protecting against allergic airway diseases. One of the plausible mechanisms by which the maturation of the infant gut microbiota would protect against allergic diseases could be by promoting the upregulation of innate immune receptors, including TLR4 and CD14, which would help to acquire greater sensitivity and response to microbial products, particularly to LPS.

In addition to the low ability to counteract Th2 cell responses, infant environments have been shown to produce pro-Th2 factors that contribute to the propensity to develop allergic sensitization during infancy. A hyperactive IL-33 axis has been shown to operate in the lung during the early postnatal period, increasing cytokine production by ILC2s, which in turn activates the migration and pro-Th2 function of cDC2 (152, 329, 330). This augmented IL-33 activity has been associated with the postnatal phase of lung alveolarization, suggesting that normal postnatal lung development can predispose to Th2 cell immunity. On the other hand, urbanization is linked to increased exposure to airborne particle pollution (also called particulate matter or PM). Recent studies have shown that early-life exposure to PM is associated, in many cases, with increased susceptibility to allergic sensitization, airway allergy, and asthma [reviewed in (331, 332)]. Although the exact mechanism by which PM may influence the onset of allergic disease is not yet fully understood, the airway epithelium has been shown to be the primary site for PM deposition, and it is believed that PM-driven activation of airway epithelium is central in the effects derived from PM exposure (333). The key mechanism by which PM is thought to exert its effects is the generation of reactive oxygen species (ROS), which induce antioxidant and inflammatory responses in exposed epithelial cells (334, 335). Moreover, PM can affect the integrity of the airway epithelial barrier (334). Therefore, the increased susceptibility to develop allergic disease in the presence of PM may arise from impaired barrier function of the epithelium, leading to increased permeability of the airway mucosa to allergens, and increased PM-stimulated release of alarmin cytokines that promote type-2 immunity to allergens (333). Still, more studies are needed to identify the exact mechanisms by which PM may be a risk factor for allergic diseases, particularly during childhood. Furthermore, the critical age window for exposure has yet to be established. In conclusion, both the lack of inhibitory signals and the increase in triggering factors contribute to augmented type 2 immunity and allergen sensitization during infancy (Figure 3). The onset of allergic sensitization coincides with the early establishment and evolution of the gut, lung, and skin microbiome and the structural development of the lungs in early childhood. Additionally, it is influenced by dietary behaviors and environmental exposures, including biological and chemical contaminants. How these factors may contribute to the risk of developing Th2 responses early in life and the underlying mechanisms remain to be fully explored. Obtaining this knowledge would help establish interventions and recommendations that seek to prevent sensitization to airborne allergens in the first years of life.

Finally, it is important to note that although exposure to highly LPS-contaminated inhalant allergens protects from the development of allergic Th2 cell responses during infancy and childhood, high endotoxin exposure can be a respiratory hazard in adulthood (336). The airway response in adults exposed to dust containing high levels of LPS is generally characterized by increased production of the pro-inflammatory cytokines IL-1, IL-6, and TFNα, the development of Th1 and Th17 responses, and infiltration of neutrophils (337–343). Overall, although hyporesponsiveness to LPS during infancy makes high LPS exposure necessary to prevent Th2 allergic responses, increased sensitivity to LPS with age implies that same high exposure to LPS may be detrimental by inducing an excessive pro-inflammatory response. Thus, hygienic perspectives should be different in childhood and adulthood and according to the degree of sensitivity to endotoxin.

## Concluding remarks

The early window of life is particularly predisposed to developing Th2 cell responses after initial contact with aeroallergens. This appears to be due to an imbalance between signals that counteract and promote Th2 cell development. cDCs elicit Th2 cell responses after a complex interaction with epithelial cells, monocytes, ILC2, and probably other immune cells that occur in the context of exposure to allergens harboring protease activity and PRR ligands. Additionally, it is now recognized that the succession of microbiota in early life and the exposure to chemical and biological contaminants make essential contributions to the infant response to allergens by altering intercellular communications. In the future, it will be crucial to understand how the infant microenvironment controls the specific signals delivered upon encountering an allergen and how these are influenced by diet and different environmental exposures, ultimately allowing us to understand better how all these signals shape the functionality of the cDCs and the specific T cell responses elicited.
